# Case report: Fast disease progression during adjuvant therapy with anti-PD-1 in stage III melanoma patients

**DOI:** 10.3389/fonc.2024.1437325

**Published:** 2024-08-01

**Authors:** Francesca Romana Di Pietro, Sofia Verkhovskaia, Rosa Falcone, Giulia Poti, Maria Luigia Carbone, Maria Francesca Morelli, Albina Rita Zappalà, Roberto Morese, Zorika Christiana Di Rocco, Gabriele Piesco, Paolo Chesi, Cristina Maria Failla, Paolo Marchetti, Federica De Galitiis

**Affiliations:** ^1^ Oncology and Dermato-oncology Department, Istituto Dermopatico dell'Immacolata (IDI)-IRCCS, Rome, Italy; ^2^ Clinical Trial Center, Istituto Dermopatico dell'Immacolata (IDI)-IRCCS, Rome, Italy; ^3^ Laboratory of Experimental Immunology, Istituto Dermopatico dell'Immacolata (IDI)-IRCCS, Rome, Italy; ^4^ Scientific Direction, Istituto Dermopatico dell'Immacolata (IDI)-IRCCS, Rome, Italy

**Keywords:** melanoma, adjuvant immunotherapy, anti-PD-1, hyperprogression disease, molecular profiling

## Abstract

**Background:**

Stage III surgically resected melanoma is a disease at high risk of recurrence. Immune checkpoint inhibitors (ICIs) and the target therapy with BRAF and MEK inhibitors significantly changed the outcome of patients with metastatic melanoma and several studies have also shown their benefit in the adjuvant setting for the delay of recurrence in stage III melanoma patients. Hyperprogression disease was observed as a possible adverse response to immunotherapy in the metastatic setting, suggesting that some patients could face additional risk of progression with ICIs, although no consensus was found for the correct definition of this event.

**Case presentation:**

We describe here two cases of rapid multiorgan metastatization during adjuvant immunotherapy in patients with stage III resected melanoma. Even though it would be not accurate to define this syndrome as hyperprogression because of apparent absence of the initial disease in the adjuvant setting, we observed in these two cases the same very rapid progression after first administration of adjuvant ICIs that resulted in death of patients within two months from the starting of treatment. Both patients had NRAS mutated melanoma.

**Conclusion:**

There is an urgent need for a better understanding of the causes of these fatal outcomes and for the identification of biomarkers that would allow to select the patients before offering them an adjuvant treatment, reducing the risk of hyperprogression. From these cases, we suggest that it could be useful a particular attention in proposing ICI adjuvant treatment based on the molecular profile.

## 1 Introduction

Melanoma is the skin cancer with the highest mortality risk. Worldwide incidence of melanoma is constantly increasing, with an annual growth rate of about 4-6% in the population with fair skin and a variable incidence based on ethnicity and geographical location (1). Until five years ago, the only postoperative therapy for melanomas at high-risk of recurrence, such as stage II and III melanomas, was interferon-α, with great benefit in reducing disease relapse and augmenting overall survival (OS) only reported for ulcerated melanoma (2).

Thereafter, approval of adjuvant therapy for stage III melanoma with anti-PD-1 antibodies, pembrolizumab and nivolumab, regardless of the tumor BRAF mutational status, opened to additional treatment possibilities. Also, adjuvant nivolumab was approved for stage IV operated and disease-free melanomas. Adjuvant anti-PD-1 antibodies demonstrated a significant advantage in Recurrence Free Survival (RFS) and Distant Metastasis Free Survival (DMFS) (3–5). More recently, pembrolizumab is also available for adjuvant treatment in stages IIB and IIC melanoma patients (6).

Despite the great benefits observed with anti-PD-1 therapy in melanoma, a half of patients did not respond and experience disease progression. A pattern of progression recently identified is called hyperprogression, and can be defined as an unexpectedly rapidly progressing disease that follows immunotherapy with anti-PD-1 antibodies in metastatic cancers, characterized by fast increased tumor volume and augmented number of metastases leading to premature death (7). Currently, there is no unique interpretation of the literature regarding this disease manifestation and whether it is an independent phenomenon related to therapy or only the normal progression of the disease.

This phenomenon has rarely been described in advanced melanoma during treatment with immune checkpoint inhibitors (ICIs). In a recent study, hyperprogression was observed in 2 patients out of 142 cases of advanced melanoma treated with ICIs (8).

Here, we describe the rapid widespread of melanoma after a single administration of anti-PD-1 antibodies as adjuvant treatment in two patients affected by resected stage III melanoma. Before starting immunotherapy, both patients appeared in good clinical conditions, asymptomatic, negative for evidence of disease on staging tests, and both developed symptoms related to advanced disease, notable increase in the lactate dehydrogenase (LDH) blood levels and radiological evidence of progression after the first administration of ICI therapy. The two patients died within two months from the adjuvant immunotherapy initial administration.

## 2 Case descriptions

The first case was a 77-year-old man with stage IIIC melanoma at diagnosis (**Table 1**). Patient medical history began in February 2019 when he was subjected to excision of a back-skin lesion. Histological examination showed a nodular melanoma, infiltrating the reticular dermis, with Breslow thickness 3.8 mm and the presence of dermal satellitosis. Tumor infiltrating lymphocytes (TILs) were absent. Tumor mutation status was assessed and found to be BRAF wildtype and mutated in the NRAS gene, in Q61R.

**Table 1:** Patient clinical features before and after adjuvant immunotherapy with ICIs.

Characteristics	Clinical case n°1	Clinical case n° 2
**Gender**	Male	Male
**Age**	77 years old	44 years old
**Comorbidities**	IPB, dyslipidemia	No
**PS ECOG**	0	0
**BRAF/NRAS status**	WT/Q61R	WT/Q61X
**Stage at diagnosis**	IIIC	IIIB
**Satellitosis or/and** **in-transit metastasis**	Yes	No
**Regional lymphadenectomy**	Yes	No
**Anti-PD-1 antibody**	Nivolumab	Pembrolizumab
**Basal LDH value**	446 U/L	159 U/L
**LDH value after first ICI**	828 U/L	1512 U/L
**Onset of symptoms**	less than 30 days	less than 30 days
**Sites of disease progression**	Brain, liver, bone, lymph nodes, lung, subcutaneous metastasis	Lung, lymph nodes, liver, subcutaneous metastasis
**Time from first ICI and death**	8 weeks	7 weeks

IPB, benign hypertrophy prostate; PS ECOG, Performance Status Eastern Cooperative Oncology Group; LDH, lactate dehydrogenase; ICI, immune checkpoint inhibitor.

In consideration of the presence of satellite nodules and the evidence at clinical examination of left axillary lymph nodes increased in volume; the patient underwent staging exam such as whole-body CT-Scan with iodine contrast, that resulted negative for evidence of other disease sites. Lymph node ultrasound confirmed left axillary lymphadenopathy. Then, lymph node biopsy was performed and resulted in tissue positivity for neoplastic cells. The patient was subjected to left axillary lymphadenectomy: 17/25 lymph nodes examined were positive for melanoma metastases.

Furthermore, wide excision around the primary melanoma site showed dermo-epidermal localization of neoplastic cells. In June 2019, a further whole-body CT-Scan with contrast was performed which confirmed the absence of disease in other sites. The final diagnosis was cutaneous melanoma pT3b pN3c, IIIC stage according to the 8^th^ edition of The American Joint Committee on Cancer (AJCC) (9). Tumor tissue was further analyzed through molecular profiling, revealing microsatellite stability and mutations in the epidermal growth factor receptor (EGFR) and p53 genes (see **Supplementary Data**, **Supplementary Figure S1**).

In consideration of the disease stage, adjuvant therapy with nivolumab was proposed to the patient, through Expanded Access Program (EAP) available in our center, at a dosage of 3 mg/kg every 14 days. On July 8^th^, 2019, the patient started the treatment with nivolumab, in good general conditions, with a Performance Status Eastern Cooperative Oncology Group (PS ECOG) of 0, and in the absence of symptoms of clinical relevance. The basal LDH value was 446 U/L (in a normal range between 208 – 378 U/L). On July 18^th^, 2019, the patient was subjected to a new dermal nodule excision, resulting in a melanoma *in transit* metastasis.

**Figure 1:**
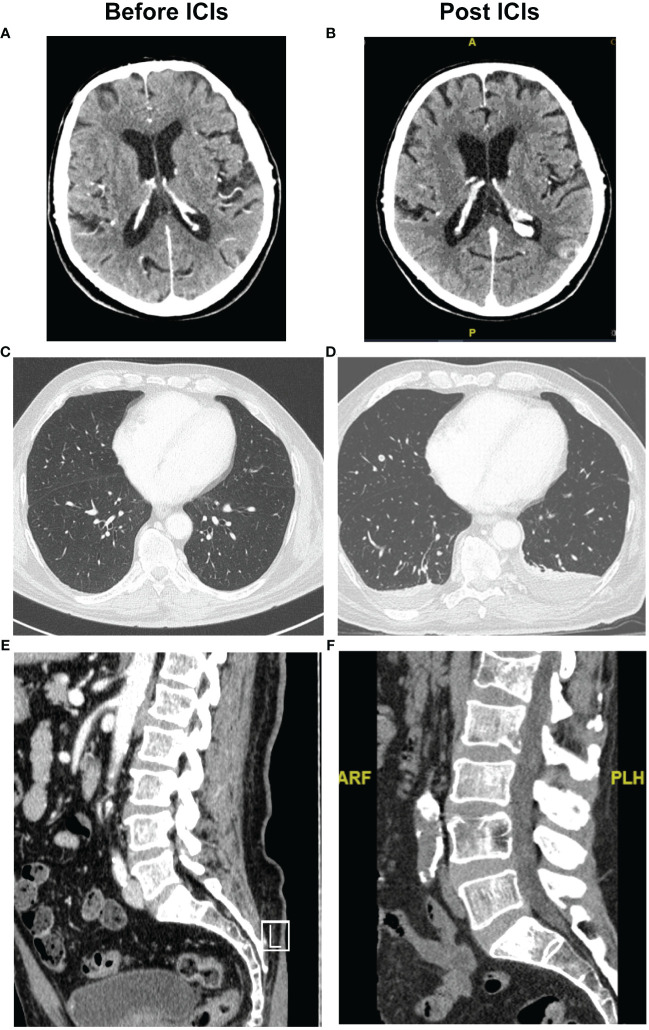
Radiographic evidence of hyperprogression after ICI of the first case. Pre-treatment with ICI, CT scan: **(A)** brain; **(C)** chest and **(E)** vertebral column. Post treatment with ICI, CT scan that showed disease progression in the same sites: **(B)** brain, **(D)** chest and **(F)** vertebral column.

At the clinical evaluation for the second administration of nivolumab on July 22^nd^, 2019, the patient reported lumbar pain. In addition, LDH value increased up to 828 U/L. On July 31^st^, 2019, the patient was hospitalized due to deterioration of the general conditions and increase of pain in the lumbar region reported Numeric Rating Scale (NRS) 8. LDH analyses was repeated giving a value of 2238 U/L. Whole body CT-Scan with contrast of the lumbosacral column and pelvis was performed, showing the appearance of multiple lesions in all the bone segments and collapse of the 2° lumbar vertebra with cortical interruption (**Figures 1A, C, E**). A new whole-body CT-Scan on August 6^th^, 2019, was then performed and showed appearance of multiple metastases: cerebral lesions in the occipital and temporal area, multiple pulmonary nodules, subcutaneous nodules, and lymph nodes increased in volume in the left axillary, homolateral pectoral and, in the mesentery sites, hepatic metastases, lesion in the right kidney and further nodule in perirenal fat (**Figures 1B, D, F**). The numerous osteolytic lesions were confirmed, interesting ribs, dorsal, lumbar, and sacral column, pelvis, and right homer. During hospitalization, the patient underwent supportive care, positioning of an orthopedic bust and was evaluated for palliative radiotherapy for bone pain. Patient clinical condition rapidly deteriorated, presenting PS ECOG of 3, and on September 10^th^, 2019, the patient died.

The second case was a 44-years-old man with evidence of atypical skin lesion on the back subjected to surgical excision in May 2023 and with a histological diagnosis of superficial spreading melanoma, Breslow thickness 2.2 mm, non-ulcerated, with mitotic index < 1/sq mm non-brisk TILs and without invasive perineural infiltration (**Table 1**).

**Figure 2:**
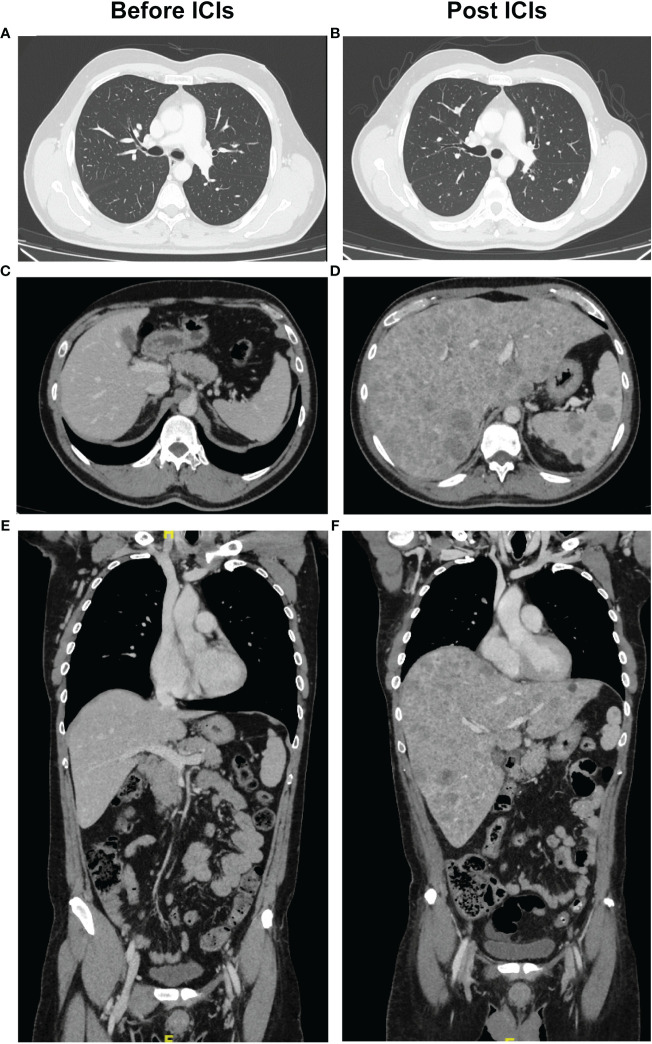
Radiographic evidence of hyperprogression after ICI of the second case. Pre-treatment with ICI, CT scan: **(A)** chest; **(C-E)** liver and spleen. Post treatment with ICI, CT scan with evidence of disease progression in the **(B)** chest and **(D-F)** liver and spleen on transverse and coronal planes.

The patient underwent whole body CT-Scan with iodine contrast and lymph node staging ultrasound, both negative for distant localizations of disease (**Figures 2A, C, E**). In consideration of the melanoma thickness, in June 2023 the patient was subjected to wide excision around the primary melanoma site, negative for satellitosis, and biopsy of the right axillary sentinel lymph node with evidence of two subcapsular metastases.

In this case, the diagnosis was of a pT3a pN1a, IIIB stage melanoma according to the 8^th^ edition of AJCC. Tumor mutation status was analyzed, resulting into BRAF wildtype and NRAS mutated in Q61X, while expression of PD-1 ligand was < 1%.

In consideration of the clinical stage and in the absence of BRAF mutation, adjuvant therapy with pembrolizumab, 200 mg every three weeks for 18 cycles, was prescribed (3). The basal LDH value was 159 U/L, lower than the normal range of 208-378 U/L. On August 7^th^, 2023, the patient underwent the first administration of pembrolizumab.

At the beginning of September, the patient arrived in the hospital reporting diarrhea grading 3 since August 25^th^, and carrying blood tests that showed value of alanine transaminase (ALT) 182 U/L (normal range 0-49 U/L), aspartate aminotransferase (AST) 122 U/L (normal range 0-34 U/L), and LDH value increased to 1512 U/L. In consideration of the severity of the clinical conditions and to understand if the alterations were due to adverse events or another cause, the patient was hospitalized. HBV and HCV tests were performed with negative results, and a liver ultrasound showed a miliary appearance of parenchyma, indicative of disease spreading. During hospitalization, the patient underwent supportive therapy with methylprednisolone 1 mg/kg then increased to 2 mg/kg, with little clinical benefit, therefore, the gastrointestinal symptoms were attributed to disease progression.

Blood tests on September 14^th^ showed a further worsening of liver function, with values of ALT 582 U/L and AST 1096 U/L, and a further increase in LDH to 4500 U/L.

On September 16^th^, whole body CT-Scan with iodine contrast was performed, which highlighted multiple bilateral pulmonary nodules, mediastinal and right axillary pathological lymphadenopathies, dermal nodules and pathological subversion of the liver and spleen (**Figures 2B, D, F**). A liver biopsy was performed which confirmed melanoma spreading and on September 23^rd^, 2023, the patient died of severe hepatic failure.

## 3 Patient perspective

The choice of adjuvant treatment in a radically operated patient requires careful evaluation in terms of risk/benefit, particularly in terms of side effects. With the introduction of immunotherapy with ICIs in adjuvant treatment setting, it must be also considered a possible worsening effect of the ICI therapy on progression of the disease and on prognosis of the patient. To give to the patients’ better indication about the adjuvant treatment, a deeper analysis of the molecular characteristics of the hyperprogression phenomenon should be done.

## 4 Discussion

In this report, we presented two cases of rapid multiorgan metastatization during adjuvant immunotherapy in patients with stage III resected melanoma.

In the first case, satellitosis was also present, in addition to disease positivity in the lymph nodes, and the patient was subjected to left axillary lymphadenectomy, all indicative of a bad prognosis. However, before starting anti-PD-1 adjuvant treatment, both patients had, as clinical characteristics, basal LDH value not significantly different from the normal range, staging whole body CT-Scan negative for metastasis, absence of symptoms suspicious of the disease, and PS ECOG of 0. Another common condition between the two patients was the tumor presence of NRAS mutated at the Q61 site. The presence of the NRAS mutation was not evaluated in the population during studies on effectiveness of adjuvant immunotherapy with ICIs in cutaneous melanoma. NRAS mutations have been described in about 15-20% of melanoma patients, mutually exclusive with BRAF mutations, except in rare cases. Mutated NRAS melanomas result in a more aggressive disease with a poorer outcome than the counterpart without mutation (10) and the best treatment for this subset of patients is still unclear. Despite the aggressiveness linked to this alteration, other studies showed a similar or better response to immunotherapy in patients carrying the NRAS mutated tumors compared to NRAS wildtype melanomas (10, 11). However, all these studies were performed in the metastatic melanoma setting, and it is not possible to verify whether NRAS mutation at the Q61 site had a role in the rapid deterioration of clinical conditions observed in our two patients.

In the literature, besides immunotherapy, hyperprogression has been reported for chemotherapy and targeted therapy treated patients as well. Whether hyperprogression could be boosted by ICI therapy has not arrived at a consensus yet (12, 13). The most frequent criterion to define hyperprogression disease was an increase up to ≥ 2-fold of the tumor growth rate during anti-PD-1 therapy leading to early death mostly in the first 2 months of treatment (14). It is not possible to exclude that such a disease course could be only due to the individual tumor growth kinetics. However, progression is generally accompanied by a gradual augmentation of the disease load and of the LDH serum amount, and death occurred not earlier than four to six months from the beginning of the immunotherapy (7, 15).

Regarding the mechanisms by which ICI therapy could favor hyperprogression (15, 16), the most probable is that ICI treatment, in some patients, has the potential to stimulate regulatory T cells (Tregs) and induce polarization of M2 macrophages and dendritic cells that produce high amounts of immunosuppressive cytokines (17, 18).

A role for the interaction of the therapeutic antibodies with the receptor expressed on macrophages had also been proposed and demonstrated in a mouse model (19).

Possible clinical factors involved in hyperprogression disease could be a high number of metastatic sites, elevated LDH levels, and high neutrophil to lymphocyte ratio (14, 20). Beyond clinical characteristics, other molecular predictive factors have been proposed, such us the microsatellite stability that identifies tumors less responsive to anti-PD-1 treatment because microsatellite instability is associated with a higher tumor mutation burden, as a result of DNA mismatch repair deficiency and present a greater lymphocyte infiltrate (21, 22).

In fact, a greater number of tumor mutations and neoantigen expression correlate with a greater response rate to ICIs. Indeed, one of the two patients we reported here showed microsatellite stability. Alterations in the EGFR are associated with poor prognosis and with an increased rate of tumor growth after treatment with anti-PD-1 agents in metastatic disease (23). An explanation for ICI resistance of the EGFR mutated tumors could be the upregulation of expression of the checkpoint molecules and their receptors that favors tumor immune escape (23). Studies in murine melanoma models showed that activation of the EGFR pathway can lead to suppression of the immune responses by stimulating Tregs. In addition, high levels of exhausted T lymphocytes have been observed in lung tumors with EGFR mutations, also promoting immune escape (24).

TP53 is a tumor suppressor gene involved in the regulation of the cell cycle and DNA repair processes. Mutations that determine TP53 loss of function are among the determinants of tumor development and aggressiveness. However, the role of TP53 in the regulation of anti-tumor immune responses is not yet completely understood. Among the possible consequences of p53 alterations have been observed the recruitment of suppressive myeloid cells and of Tregs with a negative modulation of the activity of CD8+ and CD4+ T lymphocytes (25).

Interestingly, melanoma tissue from patient 1 was reported to be mutated in both EGFR and TP53 genes. It was not possible to perform a similar analysis for patient 2 because the primitive melanoma was not treated in our Institute.

MDM2/4 amplification is found in about 7% of cancers and leads to inhibition of p53. MDM2/4 amplification was found to be related to hyperprogression disease, even if the molecular mechanism is unclear (23).

In another retrospective study on patients with advanced NSCLC progressed during immunotherapy, presence of mutations in the KRAS and serine/threonine kinase 11 (STK11) genes was specifically identified in the cohort of patients with hyperprogression (26). We could not detect MDM2/4 amplification or KRAS and STK11 mutations in our two patients.

Hyperprogression during ICI therapy was not widely investigated in melanoma. A recent multicenter study evaluated the occurrence of hyperprogression disease in metastatic melanoma patients treated with ICI, BRAF/MEK inhibitors or chemotherapy. Hyperprogression appears to be a rare event in melanoma and occurs mainly in patients with aggressive metastatic disease and high serum LDH levels (8, 13). In this analysis, a number of metastatic sites exceeding three, and liver metastasis, were identified as risk factors for hyperprogression disease (13).

Only one case of hyperprogression disease after ICI administration in the adjuvant setting has been reported (27), and disease progression was similar to that of the two patients reported here.

Altogether, these considerations suggest the necessity of selecting patients for immunotherapy with ICI in the treatment of metastatic disease in order to reduce the risk of hyperprogression.

Differently from the treatment of the metastatic melanoma, where the ratio between risks and benefits of immunotherapy with ICI is in favor of the benefits, at least in the presence of BRAF wildtype tumors, the same risks/benefits balance could be completely altered in the adjuvant setting. In fact, the data here reported suggests that there could be a group of patients for whom ICI adjuvant therapy may be hazardous.

For these patients, it is difficult to assess tumor molecular characteristics and the presence of micro metastases, not detected by common imaging methods, that could indicate an increased risk of hyperprogression. A promising tool to overcome this aspect could be the analysis of circulating tumor DNA (ctDNA), the fraction of free DNA in the bloodstream that is released from cancer cells due to cell death (28).

In the COMBI-I clinical trial, ctDNA analysis from blood samples taken at baseline and during treatment revealed that metastatic melanoma patients achieving complete responses had lower ctDNA levels at baseline compared to those with partial responses or stable disease (29).

Moreover, genome instability number of ctDNA could be a circulating biomarker of hyperprogression (30).

Recently, adjuvant ICI therapy has also been approved in other pathologies, particularly pembrolizumab for kidney and breast tumors, and atezolizumab in NCSLC (31–33). Therefore, there is an urgent need to identify molecular markers of hyperprogression risk in order to characterize each tumor deeply, before suggesting to a patient an adjuvant treatment without the risk of worsening effects in patients initially defined as disease-free.

## Data availability statement

The datasets presented in this article are not readily available because data are available upon request. Requests to access the datasets should be directed to marialuigia.carbone@idi.it.

## Ethics statement

The studies involving humans were approved by Institute Ethical Committee (n. 510/3, 2018) of IDI-IRCCS, Rome, Italy. The studies were conducted in accordance with the local legislation and institutional requirements. The participants provided their written informed consent to participate in this study. Written informed consent was obtained from the participant/patient(s) for the publication of this case report.

## Author contributions

FP: Conceptualization, Data curation, Methodology, Writing – original draft, Writing – review & editing. SV: Data curation, Writing – original draft, Writing – review & editing. RF: Methodology, Writing – review & editing. GiP: Methodology, Writing – review & editing. MC: Data curation, Methodology, Writing – review & editing. MM: Supervision, Writing – review & editing. AZ: Supervision, Writing – review & editing. RM: Supervision, Writing – review & editing. ZR: Supervision, Writing – review & editing. GaP: Data curation, Writing – review & editing. PC: Data curation, Writing – review & editing. CF: Funding acquisition, Writing – original draft, Writing – review & editing. PM: Conceptualization, Funding acquisition, Writing – review & editing. FG: Funding acquisition, Writing – review & editing, Conceptualization.
