# Supplementary material for: Case report: Fast disease progression during adjuvant therapy with anti-PD-1 in stage III melanoma patients
Source: Front Oncol. 2024 Aug 1;14:1437325. doi: 10.3389/fonc.2024.1437325 (PMC11324500; doi:10.3389/fonc.2024.1437325)
Supplement: Supplementary file 1 [file DataSheet_1.docx]

Supplementary Material

**Fast disease progression during adjuvant therapy with anti-PD-1 in stage III melanoma patients**

First Author: Francesca Romana Di Pietro, Co-Authors Sofia Verkhovskaia, Rosa Falcone, Giulia Poti, Maria Luigia Carbone*, Maria Francesca Morelli, Albina Rita Zappalà^1^, Roberto Morese, Zorika Christiana Di Rocco, Gabriele Piesco, Paolo Chesi, Cristina Maria Failla, Paolo Marchetti, and Federica De Galitiis.

*** Correspondence:** Corresponding Author: marialuigia.carbone@idi.it

# Supplementary Figure S1

**
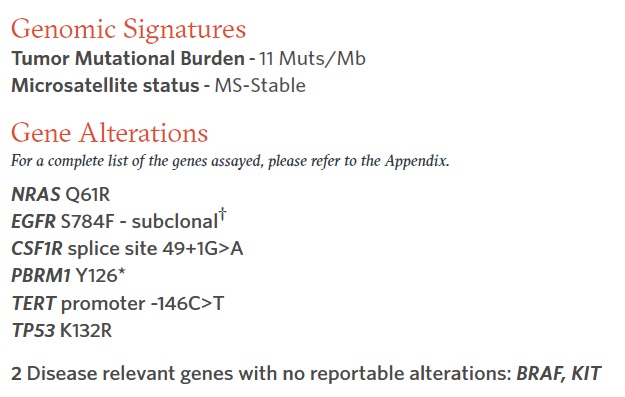
**

**Supplementary Figure S1.** Molecular profiling of first case: evidence of microsatellite status stable, alterations on EGFR and p53 genes. NRAS Q61R mutation is confirmed.
